# Genomic Determinants of Homologous Recombination Deficiency across Human Cancers

**DOI:** 10.3390/cancers13184572

**Published:** 2021-09-12

**Authors:** Tao Qing, Xinfeng Wang, Tomi Jun, Li Ding, Lajos Pusztai, Kuan-Lin Huang

**Affiliations:** 1 Breast Medical Oncology, School of Medicine, Yale University, New Haven, CT 06511, USA; tao.qing@yale.edu; 2 Department of Thoracic Surgery, National Cancer Center/National Clinical Research Center for Cancer/Cancer Hospital, Chinese Academy of Medical Sciences and Peking Union Medical College, Beijing 100021, China; 1w2x3f2012@gmail.com; 3 Division of Hematology and Medical Oncology, Tisch Cancer Institute, Icahn School of Medicine at Mount Sinai, New York, NY 10029, USA; Tomi.Jun@mountsinai.org; 4 Department of Medicine, McDonnell Genome Institute, Washington University in St. Louis, St. Louis, MO 63110, USA; lding@wustl.edu; 5 Department of Genetics, Siteman Cancer Center, Washington University in St. Louis, St. Louis, MO 63110, USA; 6 Department of Genetics and Genomic Sciences, Tisch Cancer Institute, Icahn Institute for Data Science and Genomic Technology, Icahn School of Medicine at Mount Sinai, New York, NY 10029, USA; 7 Center for Transformative Disease Modeling, Tisch Cancer Institute, Icahn Institute for Data Science and Genomic Technology, Icahn School of Medicine at Mount Sinai, New York, NY 10029, USA

**Keywords:** DNA damage repair, homologous recombination, germline and somatic, copy number variation

## Abstract

**Simple Summary:**

Tumors with homologous recombination deficiency (HRD) respond to Poly-ADP ribose polymerase inhibitor (PARPi) therapy in breast, ovarian, prostate, and pancreatic cancers. However, in addition to a handful of known pathogenic variants including those affecting *BRCA1/2*, it remains unclear what other genomic events can cause HRD. Our study systematically examined the germline and somatic genome of over 9000 cancers. We identified alterations associated with HRD, including mutations in *ATM* and *ATR* genes; specific deletions in stomach, bladder, and lung cancer; and *BRCA*-wild type breast, ovarian, and pancreatic cancers. These findings suggest a potentially broader utility for PARPi for cancers harboring a wide range of genomic alterations.

**Abstract:**

Germline *BRCA1/2* mutations associated with HRD are clinical biomarkers for sensitivity to poly-ADP ribose polymerase inhibitors (PARPi) treatment in breast, ovarian, pancreatic, and prostate cancers. However, it remains unclear whether other mutations may also lead to HRD and PARPi sensitivity across a broader range of cancer types. Our goal was to determine the germline or somatic alterations associated with the HRD phenotype that might therefore confer PARPi sensitivity. Using germline and somatic genomic data from over 9000 tumors representing 32 cancer types, we examined associations between HRD scores and pathogenic germline variants, somatic driver mutations, and copy number deletions in 30 candidate genes involved in homologous recombination. We identified several germline and somatic mutations (e.g., *BRCA1/2*, *PALB2*, *ATM*, and *ATR* mutations) associated with HRD phenotype in ovarian, breast, pancreatic, stomach, bladder, and lung cancer. The co-occurrence of germline *BRCA1* variants and somatic *TP53* mutations was significantly associated with increasing HRD in breast cancer. Notably, we also identified multiple somatic copy number deletions associated with HRD. Our study suggests that multiple cancer types include tumor subsets that show HRD phenotype and should be considered in the future clinical studies of PARPi and synthetic lethality strategies exploiting HRD, which can be caused by a large number of genomic alterations.

## 1. Introduction

Tumor cells with homologous recombination deficiency (HRD) are vulnerable to the inhibition of the DNA damage repair mechanism mediated by poly-ADP ribose polymerase (PARP). PARP inhibitors (PARPi) leverage this synthetic lethality and are US FDA approved to treat germline *BRCA1* or *BRCA2*-mutant breast, ovarian, pancreatic, and prostate cancers [1,2,3,4,5]. Clinical trials have also demonstrated that somatic *BRCA1/2* and germline *PALB2* mutations, as well as HRD determined by the myChoice^®^ CDx HRD assay (Myriad Genetics Inc., Salt Lake, UT, USA) can serve as biomarkers of PARPi response [2,6,7]. Notably, pre-clinical studies that investigated PARPi sensitivity showed a broader range of activity than its current clinical use across cancer types including gastrointestinal and genitourinary cancers [8,9,10]. These observations suggest that genomic alterations in genes involved in homologous recombination (HR) other than *BRCA1/2* could also cause HRD [2,7] and implicate new treatment opportunities.

Many genes are involved in the homologous recombination (e.g., *BRCA1/2*, *PALB2*) and DNA damage sensing (e.g., *ATM*, *ATR*, *CHEK2*) and therefore regulate HR, which we refer to as “HR genes” herein. The function of HR genes can be disrupted through inherited germline variants, acquired somatic mutations, epigenetic silencing, and somatic copy number variations. Approximately 5% of all cancers represented in The Cancer Genome Atlas (TCGA) carry germline pathogenic variants in HR genes [11,12], and cancers also frequently harbor somatic mutations or show copy-number deletions [13]. These genetic alterations are expected to disrupt DNA repair function. However, their relative contribution to shaping HRD and their potential as biomarkers for PARPi sensitivity remain to be determined.

Here, we systematically determined the associations between HRD phenotypes and pathogenic germline variants, somatic driver mutations, and somatic copy number deletions of 30 HR genes across 32 cancer types. Using the HRD phenotype score captured by whole-exome sequencing and SNP array data from over 9000 cases in TCGA cohort, we identified cancer type specific associations between germline and somatic mutations in each HR gene with the HRD phenotype. We further utilized a structural equation modeling method to estimate the combined germline or somatic contributions and described how germline variants may collaborate with specific somatic drivers. We also identified novel somatic copy number deletions that are associated with the HRD phenotype. Altogether, these findings provide a catalog of genomic alterations that may lead to the HRD phenotype and serve potential biomarkers for PARPi treatment.

## 2. Materials and Methods

### 2.1. Patients

We obtained germline variants and somatic mutations from 10,389 and 10,295 cases, respectively including 10,080 samples with both types of data. We excluded the 342 hypermutators and 226 microsatellite instability (MSI) high tumors from the 10,080 cases, resulting in 9512 cases that were included in our mutation analyses (Figure S1 and Table S1).

We collected gene-level and arm-level copy number variation (CNV) events from 10,713 and 10,399 cases, including 10,149 samples with both types of date. We excluded 561 cases without HRD score and 252 cases without somatic mutation data from 10,149 cases, resulting in 9336 cases included our CNV analyses (Figure S1 and Table S1). 

### 2.2. Genomic Datasets of TCGA Patients

TCGA HRD score: We obtained the pre-calculated HRD scores from Knijnenburg et al. [13]. The HRD score is constructed from three types of CNV [13]: (i) binary deep deletions from PanCanAtlas GISTIC2.0 analysis corresponding to GISTIC calls of “−2” which indicate loss of more than half of baseline ploidy [14]; (ii) CNV burden scores generated by TCGA PanCanAtlas Aneuploidy study using ABSOLUTE and SNP-array data [15]; and (iii) segment loss-of-heterozygosity (LOH) scores that capture the total number of segments with LOH [15,16]. The Knijnenburg et al. HRD score is calculated as the sum of the number of loss-of-heterozygosity in the genomic region of *BRCA1/2* [15,16], telomeric allelic imbalance [17], and large-scale state transitions across the entire genome region [18]. The resulting HRD score is conceptually similar to the clinically available MyChoice DCx assay.

TCGA germline cancer predisposing variants: we obtained 853 germlines pathogenic/likely pathogenic variants that were identified from 10,389 patients. Germline variants were downloaded from The Genomic Data Commons database (GDC, https://gdc.cancer.gov/about-data/publications/PanCanAtlas-Germline-AWG (accessed on 11 August 2021)) [12,19].

TCGA somatic mutations: all somatic mutations of 10,295 cancers were obtained from the Multi-Center Mutation Calling in Multiple Cancers (MC3) dataset [20]. Tumor mutation burden (TMB) was calculated as the total number of all somatic mutations in all human genes. The pre-calculated functional prediction results of all somatic mutations and the designation of “likely driver mutation” for 299 genes were taken from TCGA PanCanAtlas Driver study which used [21]. The likely somatic driver mutations included truncating mutations, frameshift and in-frame shift indels, nonsense, splice site, and translation start site mutations, or missense mutations predicted as deleterious.

TCGA somatic copy number variation data: we obtained arm-level and gene-level CNV data from the PanCanAtlas Aneuploidy study (https://gdc.cancer.gov/about-data/publications/pancanatlas, accessed date 11 August 2021) [15]. The acute myeloid leukemia (LAML) data is not available, thus LAML was excluded from the CNV analysis. The arm-level and gene-level events indicate that the copy number gain/loss effect an entire chromosome arm or a specific genomic region that encodes gene. CNV was assessed with Affymetrix SNP 6.0 arrays (Santa Clara, CA, USA) [15] and gene-level CNV values were generated by GISTIC [14]. GISTIC calls of “−2” and “2” which indicate a loss or gain of more than half of baseline ploidy were assigned as deep deletions or amplification, respectively. Chromosome arm-level events were determined using the ABSOLUTE algorithm [16]. Only deep deletions (−2) were considered for association analysis in our study.

Hypermutators: we designated 344 TCGA cases hypermutated based on Bailey et al. [21]. The hypermutators were defined as samples with mutation burden greater than 1.5 times the interquartile range above the third quartile in their respective cancer types, and the number of mutations in a sample exceeds 1000 [21].

MSI: as a measure of MSI, we took the MSIsensor scores which were derived from standard tumor-normal paired sequence data from Niu et al. [22]. MSI cases were defined as those with an MSIsensor score > 4 [22] according to the TCGA PanCanAtlas [21].

Genetic principal components of TCGA cohort: given that the ancestral genetic background may influence genomic alterations, we obtain the pre-calculated principal components (PCs) from the WashU genetic ancestry analysis of the TCGA PanCanAtlas project [12,23]. The PCs were calculated on 298,004 variants with MAF > 0.15 and low missingness, and PC1 and PC2 accounted for 51.6% and 29.2% of the variations across the first 20 PCs [23] and were included as covariates in the regression analysis.

### 2.3. Association Analyses of Germline and Somatic Mutations Using Multivariate Regression Models

We use a linear regression model to estimate the influence of germline or somatic alterations on the HRD with the “glm” function of the “base” package of the R-project [24]. Since the HRD may be affected by age and genetic background, the analysis was controlled for covariates, including patients’ age at diagnosis and population substructure (first two PCs) [23]. The model is:

$$HRD\;\sim Germline,somatic\;alteration\;\left(0,1\right)+Age+PC1+PC2$$

Only genes with predisposing variants harbored at least 4 individuals within the cancer cohort were included in the regression analysis. We perform this analysis within each cancer type.

### 2.4. Association Analyses of Gene-Level CNV Using Multivariate Regression Models

We used a linear regression model to estimate the influence of gene-level CNV events on the HRD with the “glm” function of the “base” package of the R-project. Given that the copy number variation may be affected by age, gender, arm-level CNV, the analysis was controlled for covariates, including patients’ age at diagnosis, gender, and arm-level CNV events, as well as TMB and the first two PCs [15,23,25]. The model is:

$$HRD\;\sim Gene\text{-}level\;CNV\;events\;\left(0,1\right)+Age+Gender+TMB+Arm\text{-}level\;CNV\;events+PC1+PC2$$

We performed this analysis within each cancer type. For breast cancer and prostate cancer, we excluded gender from the covariates.

### 2.5. Independent Contribution of Germline and Somatic Mutations

We use a Partial Least Squares Path Modeling (PLS-PM) analysis to investigate the contribution of germline and somatic mutations (Figure S3). The PLS-PM is a multivariate data analysis method which introduces latent variables for analyzing systems of relationships between multiple variables [26]. We include two latent variables (germline and somatic) in our analysis. The PLS-PM algorithm includes measurement model and structural model. The measurement model represents the relationships between the individual genes and the latent variables which generates coefficient (β) denoting the contribution of mutant genes to latent variable. The structural model represents the relationships between the latent variables which also provides coefficient and *p* value measuring the relative contribution of latent variables to HRD. In our analysis, we identified that germline and somatic mutations of 30 DNA damage repair genes were associated with HRD. We only use the 9512 overlapped samples with germline, somatic, and signature data available in this analysis. We perform PLS-PM analysis with the R packages “plspm” [27]. We compared the HRD score in cases with germline, somatic, both germline and somatic HR mutations, and the wildtype cases using the two-sided Mann–Whitney test.

### 2.6. Co-Occurrence and Mutual Exclusivity of Germline and Somatic Mutations

We estimate the interaction between germline predisposing variants and somatic driver mutations by using Fisher’s exact test. We assign 0 if wild type and 1 if mutated to each sample in the TCGA for each gene at both germline and somatic levels. We perform this analysis across all samples and within each cancer types separately. We compared the HRD score in cases with germline *BRCA1*, somatic *TP53*, both germline *BRCA1* and somatic *TP53* mutations, and the wildtype cases using a two-sided Mann–Whitney test.

### 2.7. Adjustment for Multiple Comparisons

*p* values were adjusted by the Benjamini–Hochberg procedure for false discovery rate (FDR) were computed globally across all analyses by genes and cancer types. All the analyses in this study were performed using scripts written with the R programming language. The significant effects of germline variants, somatic mutations, and somatic copy number variations were defined as FDR < 0.05.

## 3. Results

### 3.1. Germline and Somatic Mutations Associated with HRD in 32 Cancer Types

**Figure 1:**
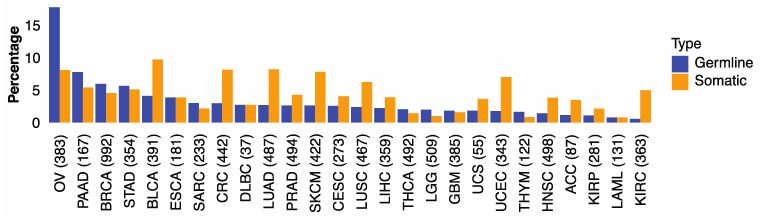
The percentage of germline and somatic Homologous Recombination mutations in 32 TCGA cancer types. The proportion of TCGA cases within each cancer type carrying germline variants (blue) or somatic mutations (orange) in 30 HR genes.

We first examined if genomic alterations in 30 genes involved with HR are associated with HRD phenotype captured by the HRD score of Knijnenburg et al. [13]. The HR genes included 21 genes in the homologous recombination pathway and nine DNA damage sensor-related genes (*ATM*, *ATR*, *ATRIP*, *CHEK1*, *CHEK2*, *MDC1*, *RNMT*, *TOPBP1*, *TREX1*) (Table S2) [28,29,30]. We found that microsatellite instability (MSI) was correlated with HRD score in several cancer types (Figure S2). To mitigate potential noise caused by large variations of genome instability in some cancers, we excluded hypermutators (*n* = 344) and microsatellite instability (*n* = 226) tumors and limited subsequent analyses to the remaining 9512 cases, comprising 32 cancer types (Figure S1, Table S1). We found that 3.5% of the 9512 cases carried germline pathogenic variants (from now on referred to as germline variants) and 4.5% harbored somatic driver mutations in HR genes. The percentages of cases carried germline or somatic HR mutations varied across cancer types: ovarian cancer (OV) showed the highest frequencies of germline variants (17.8%) and somatic mutations (8.1%), followed by pancreatic cancer (PAAD), breast cancer (BRCA), stomach cancer (STAD), and urothelial bladder carcinoma (BLCA) (Figure 1). These results illustrate the distinct germline and somatic landscapes of HR mutations across cancer types.

We next sought to identify the germline and somatic alterations that are associated with the tumors’ HRD phenotype by using an HRD score, which was previously calculated as a weighted sum of Loss of Heterozygosity (LOH), Telomeric Allelic Imbalance (TAI), and Large-scale State Transitions (LST) events in tumor of the TCGA PanCanAtlas project across 32 cancer types [13].

Using a multivariate regression model, corrected for age and genetic ancestry represented by principal components, we found 12 positive correlations between the HRD score and germline variants in five (i.e., *BRCA1*, *BRCA2*, *PABL2*, *ATM*, *ATR*) genes across cancer types (Table 1). Both, *BRCA1* and *BRCA2* variants were associated with higher HRD scores in BRCA (FDR < 1.3 × 10^−42^) and OV (FDR < 5.0 × 10^−12^), *BRCA2* (but not *BRCA1*) variants were also associated with higher HRD of PAAD (FDR = 1.3 × 10^−17^) and stomach cancer (STAD) (FDR = 6.7 × 10^−17^) (Table 1). We also found that germline *PALB2* variants are associated with higher HRD in STAD (FDR = 1.2 × 10^−41^). Germline *ATR* and *ATM* were associated with higher HRD in BRCA (FDR < 8.5 × 10^−8^), and germline *ATM* variants in prostate cancer (PRAD, FDR = 9.0 × 10^−6^), lung adenocarcinoma (LUAD, FDR = 5.8 × 10^−5^), and STAD (FDR = 0.007) (Table 1). While *BRCA1/2* germline variants in BRCA, OV, PAAD and prostate cancer are well known to be associated with HRD phenotype, our results identify a much broader range of cancers with different germline alterations in HR-related genes that are significantly associated with HRD (Table 1 and Table S3). 

We conducted a similar multivariate regression analysis for somatic mutations, and identified 24 significant associations in five (i.e., *BRCA1*, *BRCA2*, *ATM*, *ATR*, *CHEK2*) genes and higher HRD (FDR < 0.05) across cancer types. Somatic mutations of *BRCA1* and *BRCA2* were significantly associated with HRD in BRCA, OV, and BLCA, and lung squamous cell carcinoma (LUSC) (FDR < 0.002, Table 1). Somatic *BRCA2* mutations were also associated with HRD in STAD, PRAD, and colon rectum adenocarcinoma (CRC) (FDR < 0.01). Somatic mutations of *ATM* and *ATR* were associated with higher HRD in BLCA, LUAD, CRC, PRAD, head–neck squamous cell carcinoma (HNSC), BRCA, and kidney renal clear cell carcinoma (KIRC) (FDR < 0.008, Table 1). Somatic mutations of *CHEK2* significantly increased HRD in uterine corpus endometrial carcinoma (UCEC, FDR < 3.3×10^−7^, Table 1). Of note, we also identified negative associations between HRD scores and somatic HR mutations in cancer types including UCEC, LUAD, skin cutaneous melanoma (SKCM), and LUSC (FDR < 0.03, Table 1). Overall, these results highlight that both germline and somatic mutations can be associated with high HRD and the importance of different HR genes to maintain genomic stability can vary from cancer type to cancer type.

### 3.2. Relative Contributions of Germline and Somatic Mutations on HRD

**Figure 2:**
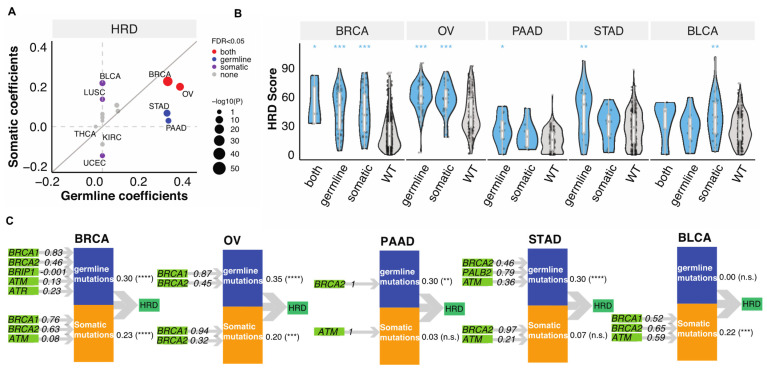
The combined contributions of germline variants and somatic mutations to HRD. (**A**). The combined germline and somatic contributions represented by coefficients obtained from the PLS-PM analysis. Each dot represents a cancer type. The size of the circles represents −log10(*p*). Grey, purple, blue, and red represent none of, somatic, germline, or both FDR meet the criteria FDR < 0.05. The grey line indicates the diagonal with slope of one. (**B**). The distribution of HRD score in combined germline and somatic carriers in BRCA (*n* = 5 (both), 53 (germline), 38 (somatic), 850 (wildtype)), OV (*n* = 66 (germline), 31 (somatic), 272 (wildtype)), PAAD (*n* = 12 (germline), 8 (somatic), 131 (wildtype)), STAD (*n* = 18 (germline), 15 (somatic), 309 (wildtype)), BLCA (*n* = 5 (both), 10 (germline), 32 (somatic), 334 (wildtype)). *p*-value was calculated using a two-sided Mann–Whitney test and FDR corrected. “***”, “**”, “*” denote FDR less than 0.0001, 0.001, and 0.05. (**C**). PLS-PM models show the significant combined contribution of germline variants and somatic mutations with FDR < 0.05 of combined effect in selected cancer types, including BRCA, OV, PAAD, STAD, and BLCA. The numbers between the latent variables and HRD indicated combined correlation coefficients identified by the PM-PLS model, the value. “****”, “***”, “**” denote FDR less than 0.00001, 0.0001, 0.001. “n.s.” denotes “not significant” (FDR > 0.05).

To delineate the relative contributions of germline variants vs. somatic mutations to the tumor HRD phenotype, we applied Partial Least Squares Path Modeling (PLS-PM) [26]. In this analysis, we only considered genes that were affected in at least four TCGA cases within a cancer type and therefore only seven genes (*BRCA1/2*, *ATM*, *ATR*, *PALB2*, *CHEK2*, *BRIP1*) were included. This may neglect the contribution of other HR genes with lower mutation frequencies, but we have limited power to identify these in the current data set. Both germline and somatic mutations significantly contributed to the HRD of BRCA (FDR < 1.5 × 10^−12^) and OV (FDR < 2.4 × 10^−4^, (Figure 2A)). In STAD and PAAD, mostly germline variants contributed to high HRD (FDR < 7.2 × 10^−4^), whereas in BLCA and LUSC high HRD were “driven” mainly by somatic mutations (FDR < 0.01, (Figure 2A)). We show the distribution of HRD scores in these cancer types by mutation status on Figure 2B.

The PLS-PM analysis also allows to quantify the relative contribution of individual gene’s to predicting higher HRD when combining all the contributors together. Both germline variants and somatic mutations of *BRCA1/2* (β > 0.32) dominantly contributed to HRD in BRCA and OV, while only germline *BRCA2* contributed to HRD in PAAD (Figure 2C). Germline *PALB2* (β = 0.79) and *BRCA2* (β = 0.46) and *ATM* (β = 0.36) variants contributed to HRD of STAD, with non-significant effects from somatic *BRCA2* (β = 0.97) and *ATM* (β = 0.21) (Figure 2C). On the other hand, somatic *BRCA1* (β = 0.52), *BRCA2* (β = 0.65) and *ATM* (β = 0.59) contributed to HRD in BLCA (Figure 2C). These results highlight that somatic and germline alterations in specific HR genes contribute differently to HRD in different cancer types.

### 3.3. Germline-Somatic Interactions Shaping HRD

**Figure 3:**
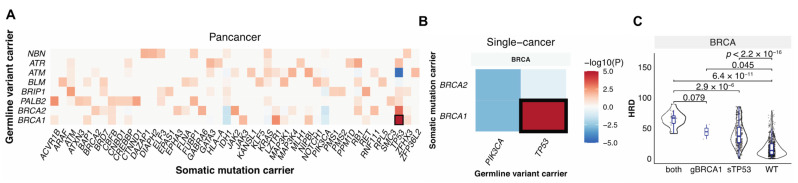
The interaction between germline pathogenic variants and somatic driver mutations. (**A**). Co-occurring germline variants and somatic mutations across all cancers. (**B**). Co-occurring germline variants and somatic mutations within each cancer type. The *x*-axis shows germline-affected genes, and the *y*-axis shows somatic-affected genes. Color denotes the Fisher exact test −log10(*p* value) showing the significance of the germline and somatic interaction. Red and blue indicate co-occurrence and mutual-exclusivity, respectively. (**C**). The distribution of HRD score in only germline (g) BRCA1 (*n* = 2), only somatic (s) TP53 (*n* = 306), and both carriers (*n* = 16). WT indicates samples without either of the mutations (*n* = 622).

Germline variants and somatic mutations can complement each other in the malignant transformation process [12,31,32], and together they can shape tumor phenotypes including HRD. To identify germline–somatic mutation pairs that may cooperate to affect HRD, we first identified the significant co-occurring germline–somatic mutation pairs considering germline variants in the 30 HR genes vs. the PanCanAtlas-defined somatic driver genes (*n* = 299) across all TCGA cancer cases and also within each cancer types using the Fisher’s exact test. Germline *BRCA1* mutations significantly co-occurred with somatic *TP53* mutations at both the pan-cancer level (FDR = 1.0 × 10^−6^, Figure 3A) and within breast cancer (FDR = 6.2 × 10^−4^, Figure 3B). We also detected trends of other germline-somatic co-occurrences, including mutually exclusive alterations between germline *BRCA1/2* and somatic *PIK3CA* (FDR = 0.2, *p* = 0.001), we found no other germline–somatic pairs that reached significance. In BRCA, samples that carried both germline *BRCA1* and somatic *TP53* mutations had higher HRD scores compared to cases with only germline *BRCA1* alteration (*p* = 0.08), or with somatic *TP53* mutation only (*p* = 2.9 × 10^−6^), or cases that were wild type for both (*p* = 6.4 × 10^−11^) (Figure 3C). These results demonstrate that while germline and somatic mutations may induce independent effects, tumors carrying both pathogenic *BRCA1* germline variants and *TP53* driver mutations show markedly increased HRD.

### 3.4. Somatic Copy Number Variations Associated with HRD

**Figure 4:**
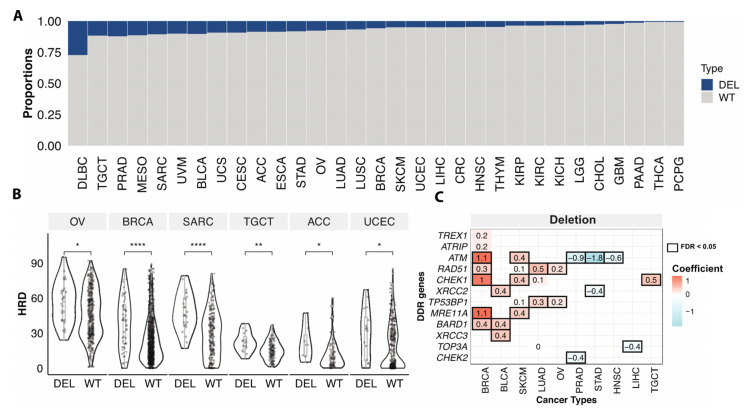
Somatic copy number deletions of HR genes associated with HRD. (**A**). The proportions of samples with somatic copy number deletion of homologous recombination and damage sensor genes in each cancer type. Blue (loss) indicates samples with somatic copy number deletions in any of 28 HR genes (excluding BRCA1/2), grey (WT) denotes non-mutated cases. (**B**). The HRD score between cases with somatic copy number deletion (DEL) of homologous recombination and damage sensor genes and wildtype (WT) cases excluding BRCA1/2 copy number variation affected cases in OV (*n* = 28 (DEL), 332 (WT)), BRCA (*n* = 53 (DEL), 860 (WT)), SARC (*n* = 23 (DEL), 193 (WT)), TGCT (*n* = 15 (DEL), 113 (WT)), ACC (*n* = 7 (DEL), 74 (WT)), UCEC (*n* = 16 (DEL), 303 (WT)). *p*-value was calculated using a two-sided Mann–Whitney test and FDR corrected. “****”, “**”, “*” denote FDR less than 0.00001, 0.001, and 0.05. (**C**). The heatmap shows the significant (FDR < 0.05, black box) association of somatic copy number deletion of individual genes with the HRD of TCGA cancer types. Both value and color in each cell represent the coefficient obtained from the multivariate logistic regression model.

Somatic copy number deletion of genes involved in HR may also compromise HR. Given that the calculation of HRD scores included copy number variation of *BRCA1/2* genes, we assessed the associations between HRD and copy number deletions focusing only on the rest of 28 HR genes. Overall, 7.0% of the non-hypermutator/microsatellite stable cases had somatic copy number deletions of genes involved in HR, excluding BRCA1 and BRCA2. Across cancer types, diffuse large B-cell lymphoma (DLBC) had the highest frequency of deletions (24.3%) in the HR genes, followed by PRAD (11.5%), and testicular germ cell tumors (TGCT) (11.7%) (Figure 4A). About 5.6% of BRCA and 7.6% of OV cases were affected by HR gene deletions (excluding BRCA1/2) (Figure 4A).

Cases with at least one somatic copy-number deletion in any of the HR genes (excluding *BRCA1/2*) showed significantly higher HRD scores compare to cases without deletions in OV, BRCA, Sarcoma (SARC), TGCT, adrenocortical carcinoma (ACC), low grade glioma (LGG), and UCEC (FDR < 0.05) (Figure 4B). Applying a multivariate regression model, we further compared the HRD scores between copy number deletion vs. wildtype cases at the gene level. Deletions of *ATM*, *RAD51*, *MRE11A*, *CHEK1*, and *BARD1* were associated with higher HRD of BRCA. We also identified other deletions associated with higher HRD in multiple cancer types, such as *XRCC2/3* and *BARD1* deletion in BLCA, *TP53BP1*, and *RAD51* deletion in OV and LUAD, and *CHEK1* deletion in TCGT and SKCM, etc. (Figure 4C). These analyses show that not only mutations but copy number deletions in HR genes can also be associated with HRD and might confer sensitivity to PARPi, warranting further mechanistic and clinical investigations.

## 4. Discussion

This study comprehensively evaluated the associations between deleterious germline variants, somatic driver mutations, and somatic copy number deletions in 30 HR-related genes and the HRD phenotype of 32 cancer types. We demonstrated that aside from *BRCA1/2* in well-known BRCA-associated cancer types (breast, ovarian, pancreatic, and prostate), several other HR gene mutations are associated with HRD in a broad range of cancers. Both germline variants and somatic mutations of HR genes can be associated with HRD phenotype. Further, their individual contributions (i.e., strength of association) and synergistic interactions between them varied by cancer type. We also demonstrated that somatic copy number deletions of HR genes are associated with HRD. These results expand the repertoire of genes and type of genomic abnormalities that may cause HRD. Further clinical studies could further validate whether these alterations could serve as potential biomarkers for treatments exploiting synthetic lethality to treat tumors with impaired HR in a broader range of cancer types.

Several PARPi have already been approved for clinical use. The US FDA approved olaparib for the treatment of germline BRCA-mutated breast, prostate, ovarian, and pancreatic cancer patients; talazoparib is also approved for germline BRCA mutated breast and ovarian cancers [33,34]. The NOVA study demonstrated that niraparib improved survival of somatic *BRCA1/2* mutant or BRCA normal but HRD positive by the Myriad myChoice CDx assay ovarian cancer [4]. The TBCRC-048 trial, demonstrated the efficacy of Olaparib in somatic BRCA mutant and germline *PALB2* mutant metastatic breast cancers [7]. Consistent with these clinical observations, our results demonstrate that either germline or somatic mutations in *BRCA1/2* genes can be associated with HRD phenotype of breast and ovarian cancer. In addition to the known associations, our data also suggests that there could be additional subsets of patients who might benefit from HR targeted therapies. For example, germline *PALB2* variants have recently been associated with familial and sporadic stomach cancer [35]. We found that germline *PALB2* variants were positively correlated with HRD in stomach cancers. Deleterious germline variants in *ATR* and *ATM* were also associated with HRD phenotype in breast cancer, *ATM* in prostate, lung, and stomach cancers, and *BRCA2* in stomach cancers.

Somatic mutations of *BRCA1/2* were also significantly associated with HRD in breast and ovarian cancers and these associations are already clinically exploited. Notably, *BRCA2* mutations were also associated with HRD in urothelial, squamous cell lung, and stomach carcinomas, where tumor response to PARPi has not yet been associated with HR mutations or HRD. We also identified previously unreported associations of somatic mutations of several other HR genes with HRD phenotype, suggesting new patient subsets that may benefit from PARPi. For example, somatic *BRCA2* and *ATM* mutations were correlated with HRD in bladder cancer, lung cancer, and colon cancer. Moreover, *ATR* mutations were associated with high HRD scores in head and neck cancer and kidney cancer (Table 1). We also noted that germline and somatic mutations contribute differently to the HRD phenotype in different cancer types. Both germline and somatic mutations significantly contributed almost equally to HRD in breast cancer. In stomach and prostate cancers, HRD was predominantly influenced by germline variants, whereas in bladder cancer somatic mutations contributed to the majority of tumors with HRD phenotype.

The effects of copy number variants on HRD and PARPi response have rarely been investigated, and only a few studies have reported *ATM*, *BRCA1*, and *BRCA2* deletions could affect sensitivity to DNA damaging chemotherapies [36,37,38]. In our analysis, we found that somatic copy number deletions in several HR genes, such as *ATM*, *RAD51*, and *CHEK1* were associated with increased HRD phenotype in OV, BRCA, SARC, and TGCT. The results suggest that somatic copy number deletion of HR genes should also be evaluated as potential biomarkers for predicting HRD and PARPi sensitivity.

One limitation of the study is that the identified associations do not imply causality, and require further experimental validation. We also recognize that results from a clinically used HRD test is not available for any of the TCGA cases. Our HRD scores were derived from the SNP array and WES data that resemble the myChoice^®^ CDx HRD assay according to the TCGA Pan-Cancer Atlas project. The HRD scores used in this study may limit the detection of HRD tumors. Further, observing an HRD phenotype may not guarantee PARPi sensitivity in tumors. Future studies using clinical cohorts with genomic and PARPi response data are needed to validate whether these tumors may be effectively treated by therapies exploiting synthetic lethality.

## 5. Conclusions

Our results showed that the HRD phenotype is associated with several genomic alterations in genes involved with homologous recombination across both BRCA-associated and non-BRCA-associated cancer types. These findings raise the possibility that many subpopulations of cancer patients might benefit from PARP inhibitors and other HR-targeted therapies.

## Figures and Tables

**Table 1:** The significant associations (FDR < 0.05) of germline variants and somatic mutations with HRD phenotype.

Index	Cancer	Gene	Mutation Type	Number of Cases Affected (%)	Correlation Coefficients	FDR
1	BRCA	*BRCA1*	germline	18 (1.8)	0.91	<1.0 × 10^−50^
2	BRCA	*BRCA2*	germline	16 (1.6)	0.63	<1.0 × 10^−50^
3	OV	*BRCA1*	germline	35 (9.1)	0.32	1.3 × 10^−42^
4	STAD	*PALB2*	germline	4 (1.1)	0.84	1.2 × 10^−41^
5	PAAD	*BRCA2*	germline	5 (3)	0.70	1.3 × 10^−17^
6	STAD	*BRCA2*	germline	4 (1.1)	0.61	6.7 × 10^−17^
7	OV	*BRCA2*	germline	26 (6.8)	0.20	5.0 × 10^−12^
8	BRCA	*ATR*	germline	5 (0.5)	0.47	7.7 × 10^−12^
9	BRCA	*ATM*	germline	9 (0.9)	0.32	8.5 × 10^−8^
10	PRAD	*ATM*	germline	6 (1.2)	0.45	9.0 × 10^−6^
11	LUAD	*ATM*	germline	6 (1.2)	0.30	5.8 × 10^−5^
12	STAD	*ATM*	germline	6 (1.7)	0.25	0.007
13	BRCA	*BRCA1*	somatic	15 (1.5)	0.79	<1.0 × 10^−50^
14	BRCA	*BRCA2*	somatic	11 (1.1)	0.81	<1.0 × 10^−50^
15	BLCA	*BRCA2*	somatic	7 (1.8)	0.56	1.6 × 10^−21^
16	OV	*BRCA1*	somatic	17 (4.4)	0.27	5.1 × 10^−17^
17	UCEC	*ATM*	somatic	15 (4.4)	−0.70	2.9 × 10^−13^
18	LUSC	*BRCA2*	somatic	7 (1.5)	0.35	6.4 × 10^−10^
19	BLCA	*BRCA1*	somatic	6 (1.5)	0.37	1.5 × 10^−8^
20	STAD	*BRCA2*	somatic	4 (1.1)	0.44	1.5 × 10^−8^
21	UCEC	*BRCA2*	somatic	7 (2)	−0.80	1.5 × 10^−8^
22	BLCA	*ATM*	somatic	23 (5.9)	0.20	1.7 × 10^−7^
23	UCEC	*CHEK2*	somatic	4 (1.2)	0.51	3.3 × 10^−7^
24	LUAD	*ATM*	somatic	24 (4.9)	0.18	1.3 × 10^−5^
25	OV	*BRCA2*	somatic	7 (1.8)	0.23	2.2 × 10^−5^
26	PRAD	*BRCA2*	somatic	5 (1)	0.42	7.8 × 10^−5^
27	CRC	*ATM*	somatic	23 (5.2)	0.20	0.0003
28	PRAD	*ATM*	somatic	15 (3)	0.24	0.0007
29	LUSC	*BRCA1*	somatic	6 (1.3)	0.21	0.002
30	LUAD	*BRCA2*	somatic	4 (0.8)	−0.46	0.004
31	HNSC	*ATR*	somatic	4 (0.8)	0.27	0.005
32	BRCA	*ATM*	somatic	14 (1.4)	0.14	0.007
33	KIRC	*ATR*	somatic	4 (1.1)	0.47	0.008
34	CRC	*BRCA2*	somatic	6 (1.4)	0.27	0.01
35	SKCM	*BRCA2*	somatic	4 (0.9)	−0.39	0.02
36	LUSC	*ATR*	somatic	4 (0.9)	−0.22	0.03

CHOL: Cholangiocarcinoma; ACC: Adrenocortical carcinoma; BLCA: Bladder Urothelial Carcinoma; BRCA: Breast invasive carcinoma; CESC: Cervical squamous cell carcinoma and endocervical adenocarcinoma; COADREAD: Colon Rectum adenocarcinoma; DLBC: Lymphoid Neoplasm Diffuse Large B-cell Lymphoma; ESCA: Esophageal carcinoma; GBM: Glioblastoma multiforme; HNSC: Head and Neck squamous cell carcinoma; KICH: Kidney Chromophobe; KIRC: Kidney renal clear cell carcinoma; KIRP: Kidney renal papillary cell carcinoma; LAML: Acute Myeloid Leukemia; LGG: Brain Lower Grade Glioma; LIHC: Liver hepatocellular carcinoma; LUAD: Lung adenocarcinoma; LUSC: Lung squamous cell carcinoma; MESO: Mesothelioma; OV: Ovarian serous cystadenocarcinoma; PAAD: Pancreatic adenocarcinoma; PCPG: Pheochromocytoma and Paraganglioma; PRAD: Prostate adenocarcinoma; SARC: Sarcoma; SKCM: Skin Cutaneous Melanoma; STAD: Stomach adenocarcinoma; TGCT: Testicular Germ Cell Tumors; THCA: Thyroid carcinoma; THYM: Thymoma; UCEC: Uterine Corpus Endometrial Carcinoma; UCS: Uterine Carcinosarcoma; UVM: Uveal Melanoma.

## Data Availability

The TCGA germline variants are available at https://gdc.cancer.gov/about-data/publications/PanCanAtlas-Germline-AWG (accessed on 11 August 2021). The TCGA somatic mutations are available at https://gdc.cancer.gov/about-data/publications/mc3-2017 (accessed on 11 August 2021). Data supporting the findings of this study are available in the Article, Supplementary Information, or from the authors upon reasonable request.

## Supplementary Materials

The following are available online at https://www.mdpi.com/2072-6694/13/18/4572/s1, Figure S1: CONSORT diagram, TCGA cases included in this study, Figure S2: The association between MSIsensor score and HRD score in all TCGA cancer cases, Figure S3: A schema of Partial Least Squares Path Modeling analysis, Table S1. TCGA samples, Table S2: 30 HR genes, Table S3: The associations of germline variants and mutations with HRD, the full list of Table 1.
