# Supplementary figures and images for: Genomic Determinants of Homologous Recombination Deficiency across Human Cancers
Source: Cancers (Basel). 2021 Sep 12;13(18):4572. doi: 10.3390/cancers13184572 (PMC8472123; doi:10.3390/cancers13184572)

**Mutation data**

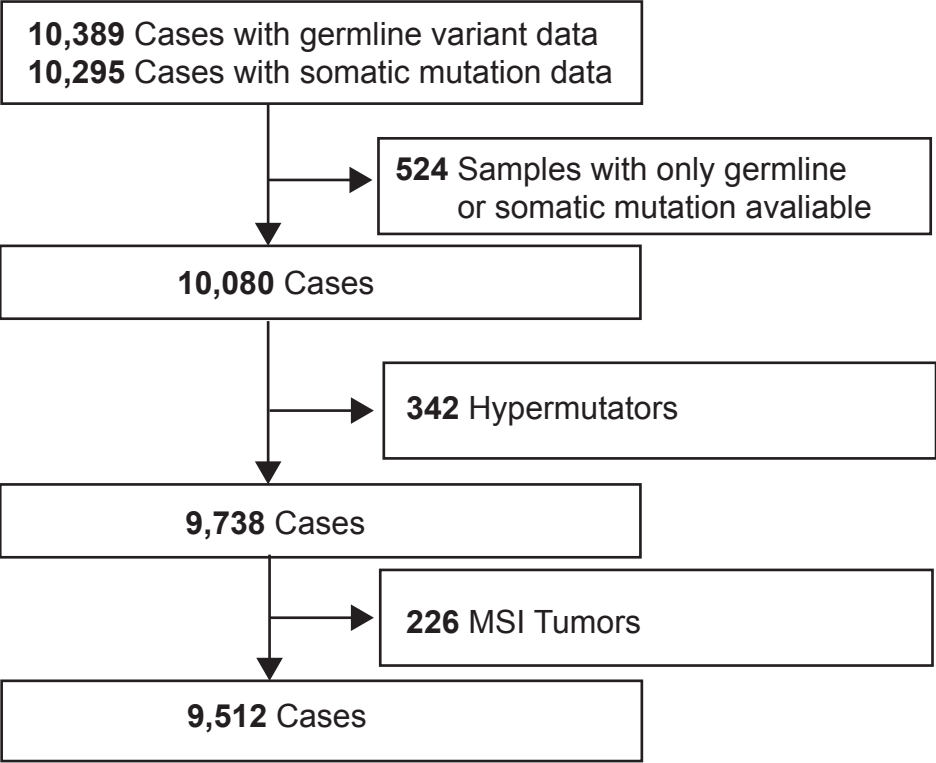

**Copy number variation data**

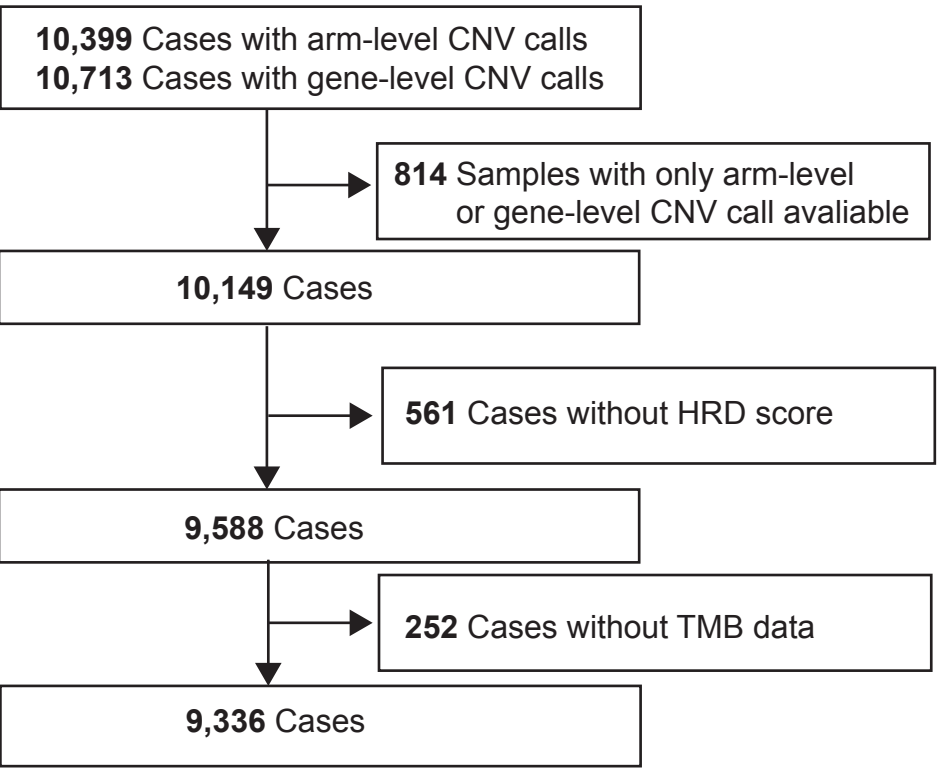

Supplement: Supplementary file 1 [file cancers-13-04572-s001.zip › Supplementary Figure1.pdf]

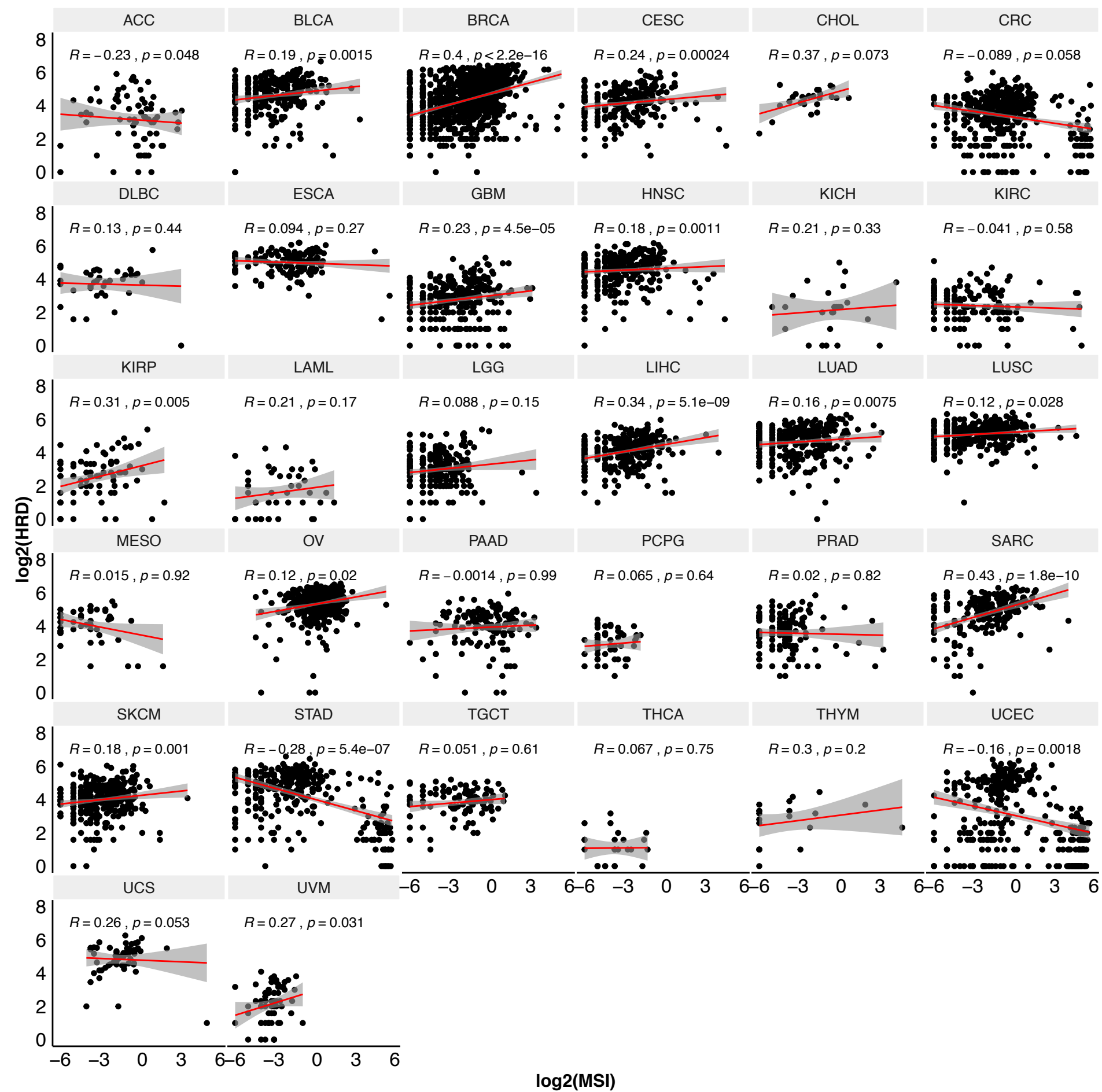

Supplement: Supplementary file 1 [file cancers-13-04572-s001.zip › Supplementary Figure2.pdf]
